# Clinical Progression of Cardiac Chronic *Trypanosoma cruzi* Infection in a Long-Term Prospective Cohort from Rural Colombia

**DOI:** 10.3390/pathogens14121198

**Published:** 2025-11-24

**Authors:** Mario J. Olivera, Julián F. Porras-Villamil, Christian Toquica-Gahona, Màrius V. Fuentes

**Affiliations:** 1 Grupo de Parasitología, Instituto Nacional de Salud, Bogotá 111321, Colombia; molivera@ins.gov.co; 2 Parasites and Health Research Group, Facultat de Farmàcia i Ciències de l’Alimentació, Universitat de València, 46010 València, Spain; 3 Department of Entomology, University of Kentucky, Lexintong, KY 40546, USA; jfporrasv@unal.edu.co; 4 Cardiology Department, Trinity Health Oakland Hospital, Pontiac, MI 48341, USA; christiantoquica@gmail.com

**Keywords:** Chagas disease, *Trypanosoma cruzi*, chronic infection, clinical progression, benznidazole, Colombia

## Abstract

Chronic *Trypanosoma cruzi* infection remains a major cause of preventable cardiomyopathy in Latin America, yet prospective data from endemic rural populations after the interruption of domestic transmission are limited. This study characterizes the long-term clinical evolution, treatment response, and cardiovascular outcomes of 80 adults with confirmed chronic infection from Mogotes, Santander, Colombia. Participants were followed from 2015 to 2023 by means of standardized clinical, electrocardiographic, echocardiographic, serological, and qPCR evaluations. The primary outcome was progression from the indeterminate to the cardiac form; secondary outcomes included worsening to advanced cardiomyopathy and cardiovascular events. During 422 person-years of follow-up (median 61.5 months), 14 participants (17.5%) experienced clinical progression (3.3 per 100 person-years) and seven (9%) died (1.7 per 100 person-years). Benznidazole treatment was initiated in 42 participants and completed by 34 (81%); only 4 of 34 (12%) remained qPCR-positive after one year, compared with 21% among untreated individuals. Progression from the indeterminate form occurred in one participant (0.7 per 100 person-years), whereas deterioration clustered among those with baseline cardiomyopathy. Despite low mortality, stage-dependent progression persisted, highlighting the need for early diagnosis, treatment adherence, and sustained molecular and cardiac monitoring in post-certification settings. These findings provide rare longitudinal evidence on chronic *T. cruzi* infection under real-world endemic conditions.

## 1. Introduction

Chagas disease, caused by the protozoan *Trypanosoma cruzi*, remains one of the most important neglected tropical diseases in the Americas [1]. Despite decades of control efforts, it continues to cause chronic cardiac damage and premature mortality, affecting an estimated 6–7 million people worldwide and constituting a leading cause of non-ischemic cardiomyopathy in Latin America [1,2]. The infection typically follows a slow and often silent course: most individuals remain in the indeterminate phase, whereas a proportion progressively develop cardiac involvement characterized by conduction disturbances, arrhythmias, ventricular dysfunction, and ultimately cardiomyopathy [3]. This chronic evolution, which can culminate in heart failure or sudden death, places Chagas cardiomyopathy among the main causes of cardiovascular mortality in endemic regions. It is important to mention that, although not as common, Chagas disease can also cause digestive and neurological manifestations [4,5].

Long-term cohort studies from Brazil and Argentina, most notably the Bambuí and Cochabamba cohorts, have provided valuable evidence on disease evolution, reporting annual progression rates of approximately 1–3% from the indeterminate to the cardiac phase [6,7,8,9,10,11]. Predictors of progression include abnormal baseline ECG findings, persistent *T. cruzi* parasitemia detected by molecular methods, and male sex [9,10,11]. However, most existing evidence derives from cohorts based on urban specialized centers in the Southern Cone, where *Rhodnius prolixus* is not the predominant vector and TcII, V, or VI lineages prevail [7,9,12]. The generalizability of these findings to other endemic settings, particularly those dominated by TcI genotypes and undergoing demographic and epidemiological transition, remains uncertain.

In Colombia, sustained vector control efforts have led to the certification of intradomiciliary transmission interruption in several historically endemic municipalities, such as Mogotes (Santander), where this community-based cohort was established [13]. As a result, a growing number of chronically infected individuals, most of whom acquired the infection decades ago, are now entering the age of highly elevated cardiovascular risk and are being diagnosed for the first time [14,15,16]. Despite these public health advances, many of these patients remain undiagnosed or untreated, facing persistent barriers to access antiparasitic therapy and long-term cardiac monitoring [17]. Furthermore, longitudinal evidence integrating molecular follow-up, clinical staging, and treatment response under real-world conditions in post-certification contexts is limited [18,19].

Closing these knowledge gaps is essential to understand how *T. cruzi* infection evolves in populations no longer exposed to active transmission but still bearing the chronic disease burden. Therefore, this study aims to characterize the long-term clinical evolution, parasitological response, and cardiovascular outcomes of chronic *T. cruzi* infection in a rural Colombian cohort under post-certification conditions. By integrating clinical, electrocardiographic, echocardiographic, molecular, and therapeutic data, this research provides new evidence on disease progression and management in endemic and post-elimination scenarios.

## 2. Materials and Methods

### 2.1. Study Design and Setting

**Figure 1:**
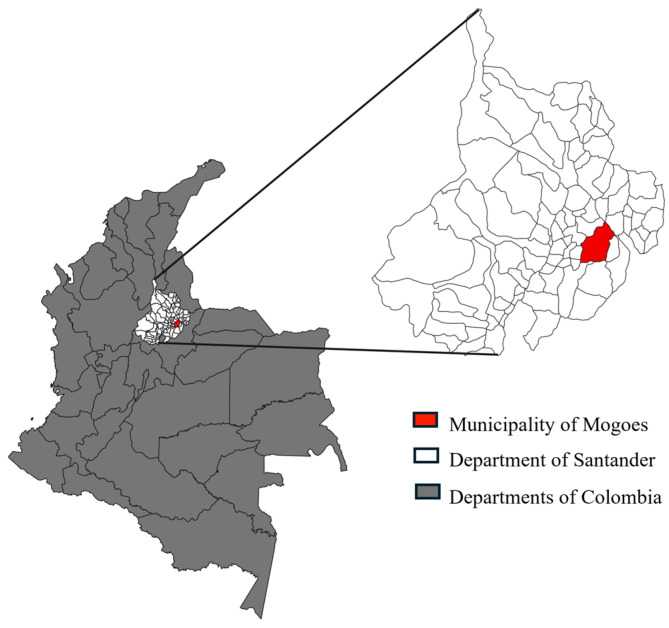
Geographic location of the municipality of Mogotes, department of Santander, Colombia.

A prospective, community-based cohort study was conducted from October 2015 to December 2023 in the municipality of Mogotes, department of Santander, northeastern Colombia (Figure 1). Mogotes is a rural Andean municipality located at approximately 1700 m above sea level, historically endemic for Chagas disease. The study adhered to the STROBE (Strengthening the Reporting of Observational Studies in Epidemiology) guidelines to ensure transparency and accuracy in reporting findings.

### 2.2. Participants

Participants were recruited through a community-based screening initiative carried out in collaboration with municipal health authorities among the general population. Eligibility criteria included being a permanent resident of the municipality, aged 18 years or older, and providing written informed consent. Individuals with positive results in two independent serological assays (enzyme-linked immunosorbent assay [ELISA] and either indirect hemagglutination or immunofluorescence [IFA]) were enrolled as confirmed cases of chronic *T. cruzi* infection.

Exclusion criteria comprised acute symptoms suggestive of recent *T. cruzi* infection, a previous confirmed diagnosis of Chagas disease or prior etiological treatment, advanced cardiomyopathy unrelated to Chagas disease, major congenital heart disease, or inability to provide informed consent.

### 2.3. Clinical and Laboratory Procedures and Outcomes

At baseline and during follow-up visits, participants underwent standardized clinical and laboratory evaluations that included detailed medical history, physical examination, 12-lead electrocardiogram (ECG), transthoracic echocardiography (ECHO), and blood sampling for molecular testing by quantitative Polymerase Chain Reaction (qPCR), performed using standardized and validated molecular diagnostic procedures [20,21]. Information on antiparasitic treatment with Benznidazole (BZN) and adherence to therapy was also recorded.

Clinical classification followed the criteria recommended by the 2nd Brazilian Consensus on Chagas Disease [22]. The indeterminate form was defined as positive serology for *T. cruzi* with normal ECG and ECHO findings and absence of symptoms compatible with Chagas cardiomyopathy. The chronic cardiac form was defined by the presence of one or more ECG abnormalities compatible with Chagas heart disease, including second- or third-degree right bundle-branch block (with or without left anterior fascicular block), frequent ventricular premature beats (more than one per ECG), polymorphic or repetitive nonsustained ventricular tachycardia, second- or third-degree atrioventricular block, sinus bradycardia with a heart rate below 50 beats per minute, sinus node dysfunction, left bundle-branch block, atrial fibrillation, electrically inactive areas, or primary ST-T segment changes. Structural cardiac alterations and left ventricular systolic dysfunction identified by ECHO were also considered compatible with the chronic cardiac form.

The severity of chronic Chagas cardiomyopathy was classified using two complementary approaches. Functional status was assessed according to the New York Heart Association (NYHA) classification, which categorizes patients as class I (asymptomatic), class II (mild symptoms with ordinary activity), class III (marked limitation of activity), and class IV (symptoms at rest). Disease stage was determined according to the Brazilian Consensus on Chagas Disease, which defines stage A as positive serology with normal ECG and ECHO, stage B1 as ECG abnormalities with preserved ventricular function, stage B2 as ECG abnormalities with mild ventricular dysfunction, stage C as symptomatic heart failure with moderate to severe dysfunction, and stage D as end-stage cardiomyopathy.

Clinical progression was defined as either the transition from the indeterminate to the chronic cardiac form or worsening of disease severity within the chronic cardiac form, evidenced by deterioration in NYHA functional class or advancement in disease stage. The primary outcome was the cumulative incidence and annual rate of clinical progression according to these definitions. Secondary outcomes included a composite endpoint of major cardiovascular events such as heart failure, stroke, device implantation (pacemaker or implantable cardioverter-defibrillator), and death.

Follow-up time was measured from the date of the first ECG assessment until the occurrence of any study outcome or the last clinical evaluation before administrative censoring in December 2023 for participants who remained under observation or were lost to follow-up.

### 2.4. Statistical Analysis

Baseline characteristics were summarized using medians and interquartile ranges for continuous variables and frequencies with percentages for categorical variables. Incidence rates of clinical progression and mortality were calculated per 100 person-years with 95% confidence intervals.

Time-to-event analyses were conducted using Kaplan–Meier estimators and Cox proportional hazards models to identify predictors of progression. Candidate covariates included age, sex, baseline clinical stage, ECG and ECHO findings, qPCR positivity, and BZN treatment. Age and left ventricular ejection fraction (LVEF) were modelled as linear effects (per 10 years and per 5% absolute change, respectively). BZN exposure was incorporated as a time-dependent covariate to account for variable treatment initiation.

Proportional hazards assumptions were verified with Schoenfeld residuals, and no major violations were detected. Multivariable models were constructed including variables with *p* < 0.20 in univariable analyses or with prior biological plausibility. Due to minimal missing data, imputation procedures were not required.

All tests were two-sided with a significance level of 0.05. Hazard ratios (HRs) with 95% confidence intervals are reported. Analyses were performed using R software (version 2025.09.1; R Foundation for Statistical Computing, Vienna, Austria). Maps were created using QGIS (version 3.22; QGIS Development Team, Open Source Geospatial Foundation Project, Beaverton, OR, USA) with official geographic data from IGAC and DANE (Colombia).

## 3. Results

### 3.1. Baseline Characteristics and Clinical Classification

Among the 112 community-screened individuals, 80 (71.4%) tested seropositive for *T. cruzi* and were enrolled in the longitudinal cohort. The study population was predominantly female (67.5%) and middle-aged (mean age 54.7 years, SD 15.7). Most participants were engaged in agricultural or domestic activities and were affiliated with Colombia’s subsidized health insurance system. Nearly all (98%) resided in rural dwellings with structural conditions favorable to triatomine infestation, and 43.8% reported having at least one relative previously diagnosed with Chagas disease (Table 1).

Comorbidities were identified in 42 (52.5%) participants, most frequently hypertension and dyslipidemia. Cardiac symptoms were reported by 35 (43.7%) participants, mainly chest pain, palpitations, and exertional dyspnea. ECG abnormalities were observed in 43 (53.7%), including right bundle-branch block, nonspecific repolarization changes, and isolated ventricular extrasystoles. ECHO alterations were detected in 17 (21.2%), predominantly mild left ventricular dilation and early systolic dysfunction (Table 1).

**Figure 2:**
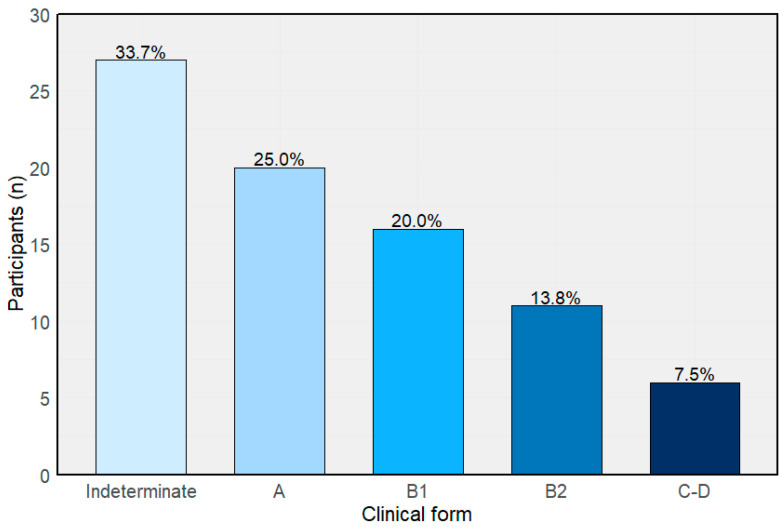
Baseline clinical classification according to the Brazilian consensus on Chagas disease.

*T. cruzi* DNA was detected by qPCR in 37 (46.2%) participants at baseline, with a median parasitemia of 1.34 parasite equivalents/mL. According to the Brazilian Consensus clinical classification, 27 (33.7%) were categorized as indeterminate, 20 (25%) as stage A, 16 (20%) as stage B1, 11 (13.8%) as stage B2, and 6 (7.5%) as stages C–D (Figure 2).

### 3.2. Clinical Outcomes and Progression

**Figure 3:**
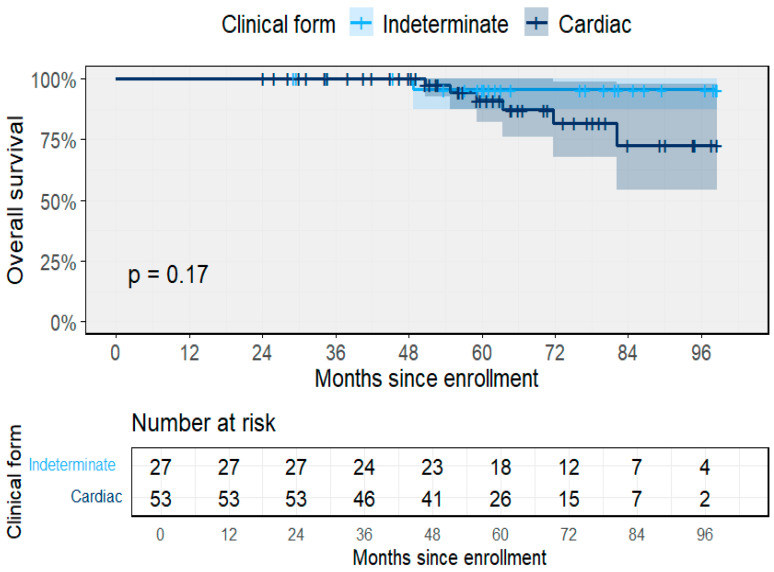
Overall survival by baseline clinical form. Kaplan–Meier survival curves stratified by baseline classification (indeterminate and cardiac). Shaded area represents the 95% CI.

Over the observation period, the cohort contributed a total of 422 person-years (PY) of follow-up, with a median duration of 61.5 months (IQR 49.1–79.5). Participants with the indeterminate form accounted for 152 PY (median 63.0 months, IQR 55.4–83.6), while those with cardiac involvement (stages A–D) contributed 270 PY (median 59.8 months, IQR 48.4–75.2). By baseline clinical stage, the median follow-up was 63.0 months for the indeterminate form, 61.1 for stage A, 56.3 for B1, 70.8 for B2, and 44.2 for C–D (Figure 3). Survival probability declined progressively across clinical forms, with lower overall survival among cardiac stages compared to indeterminate participants.

**Figure 4:**
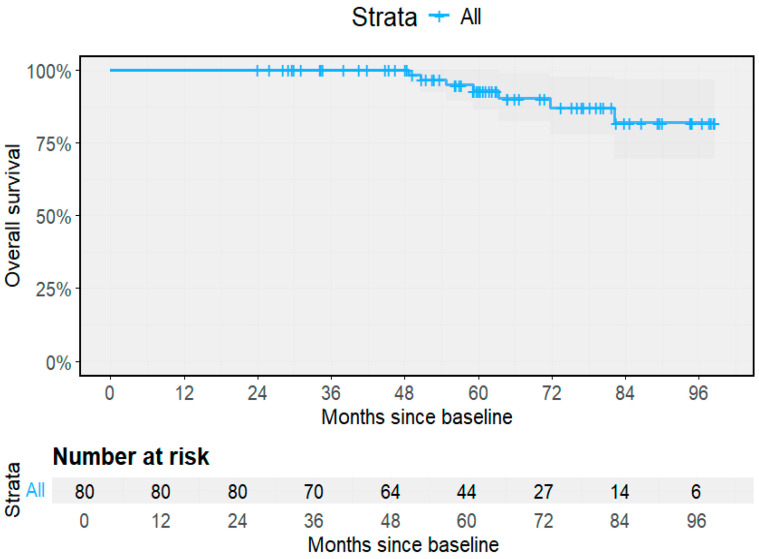
Overall survival for the entire cohort. Kaplan–Meier curve depicting survival probability during 96 months of follow-up.

A total of seven deaths were recorded during follow-up, corresponding to an overall all-cause mortality rate of 1.66 per 100 PY (95% CI 0.67–3.42). Mortality among participants with the indeterminate form was 0.66 per 100 PY (95% CI 0.02–3.66; 1/152 PY), compared with 2.22 per 100 PY (95% CI 0.82–4.84; 6/270 PY) among those with cardiac forms. One death occurred in an individual with the indeterminate form, and six occurred among participants with baseline or progressive cardiomyopathy. Median time to death was not reached in either group, reflecting the chronic nature of the infection. The overall survival curve for the entire cohort (Figure 4) shows high survival throughout the 8-year follow-up, with most deaths occurring after 60 months of observation.

**Figure 5:**
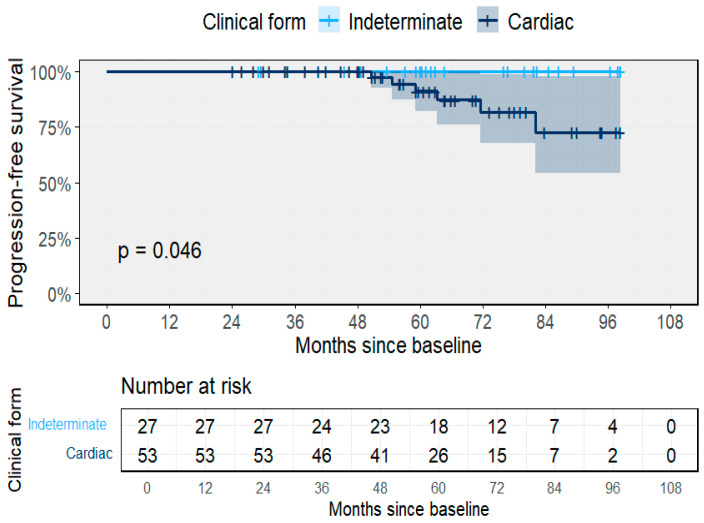
Progression-free survival by baseline clinical form. Kaplan–Meier estimates showing significantly reduced progression-free survival among participants with cardiac involvement (*p* = 0.046). Shaded area represents the 95% CI.

During follow-up, 14 participants (17.5%) experienced clinical progression, yielding an overall incidence rate of 3.3 per 100 PY (95% CI 1.8–5.7; 14/422 PY). Progression was observed in one participant with the indeterminate form (0.7 per 100 PY; 95% CI 0.02–3.7) and in 13 participants with cardiac involvement at baseline (4.8 per 100 PY; 95% CI 2.6–8.9). By baseline stage, progression rates were 0.66 per 100 PY for the indeterminate form, 2.9 for A, 1.2 for B1, 12.4 for B2, and 4.7 for C–D. The median time to clinical progression was 24.3 months among participants evolving from indeterminate to cardiac forms, 29.6 months for early cardiac stages, 41.1 months for intermediate stages, and 37.7 months for advanced stages. The probability of remaining free from clinical progression differed significantly between groups (*p* = 0.046), with lower progression-free survival among participants with cardiac involvement compared to those in the indeterminate form (Figure 5). No patient turned seronegative during the course of this study.

### 3.3. Predictors of Clinical Progression

In multivariable Cox proportional hazards models (n = 80; 14 progression events), baseline clinical stage, male sex, and age were independently associated with higher risk of clinical worsening. Compared with the indeterminate form (S0), hazards increased progressively with advancing stage. Male sex and older age were also significant predictors (Table 2).

Baseline ECG and echocardiographic abnormalities were not independently associated with progression (ECG altered: HR 0.15, 0.02–1.13; *p* = 0.067; ECHO altered: HR 0.74, 0.03–18.28; *p* = 0.850). Similarly, baseline qPCR positivity (HR 1.89, 0.47–7.54; *p* = 0.365) and LVEF per 5% absolute change (HR 1.62, 0.70–3.73; *p* = 0.255) were not significant. The proportional hazards assumption was met (global test χ^2^ = 12.8, df = 10, *p* = 0.238).

When BZN treatment was modeled as a time-dependent covariate, no significant association with progression was observed (HR 0.78, 95% CI 0.19–3.20; *p* = 0.785). The direction and magnitude of effects for other covariates remained consistent with the main model, and the proportional hazards assumption was not violated (global *p* = 0.199). Due to the limited number of deaths (n = 7), multivariable mortality models were not reported.

### 3.4. Treatment and Molecular Follow-Up

BZN was prescribed to 42 (52.5%) participants, while 38 (47.5%) did not initiate treatment due to personal reasons, reluctance to start medication, or being in advanced stages of cardiac disease. Among those who started therapy, 34 (81%) completed the full 60-day regimen, and eight (19%) discontinued treatment before completion.

Adverse events, mostly mild to moderate and appearing during the first three weeks of therapy, were reported in 29 of 42 (69%) treated participants. The most frequent adverse effects included cutaneous rash (n = 12, 28.6%), gastric discomfort or nausea (n = 9, 21.4%), pruritus (n = 5, 11.9%), headache or malaise (n = 4, 9.5%), and transient peripheral neuropathy (n = 2, 4.8%). Participants who discontinued therapy reported more intense rash or persistent gastrointestinal discomfort that interfered with daily activities. No severe adverse reactions or hospitalizations were recorded.

At one year of follow-up, qPCR was performed in all 80 participants. Among those who completed BZN treatment, 4 of 34 (11.8%) were qPCR-positive, while 30 of 34 (88.2%) were negative for detectable *T. cruzi* DNA. Among untreated participants, 10 of 38 (26.3%) had detectable *T. cruzi* DNA. Additional molecular testing beyond the first year was not performed.

Follow-up serological testing at one year showed that all participants, both treated and untreated, remained seropositive by ELISA and IFA. No cases of seroreversion were documented, and qualitative titers were unchanged from baseline.

### 3.5. Treatment Outcomes and Clinical Progression

**Figure 6:**
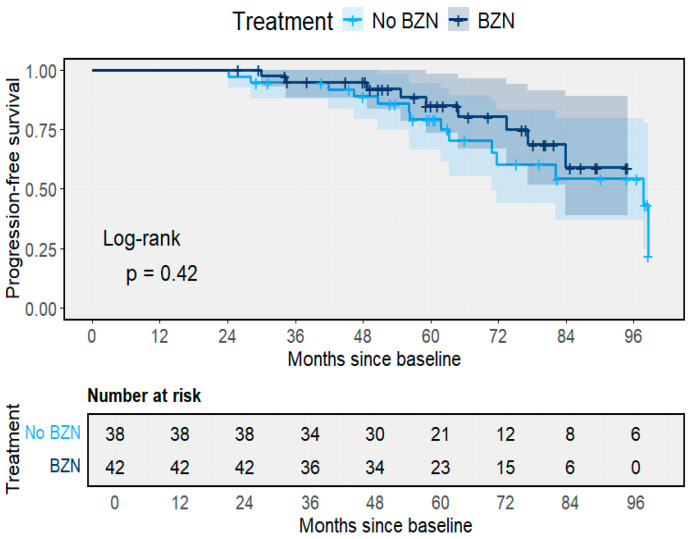
Progression-free survival according to benznidazole treatment. Kaplan–Meier curves comparing treated (BZN) and untreated participants (Log-rank *p* = 0.42). Shaded area represents the 95% CI.

The impact of BZN therapy on long-term clinical outcomes was assessed using time-to-event and competing-risk analyses. Kaplan–Meier estimates of progression-free survival demonstrated a modest, non-significant difference between treated and untreated participants (Log-rank *p* = 0.42) (Figure 6). After eight years of follow-up, progression-free survival remained slightly higher among individuals who received BZN (78.5%) compared with those untreated (73.1%). The survival curves were largely parallel, indicating that BZN exposure did not substantially alter the time to clinical progression within the study period.

**Figure 7:**
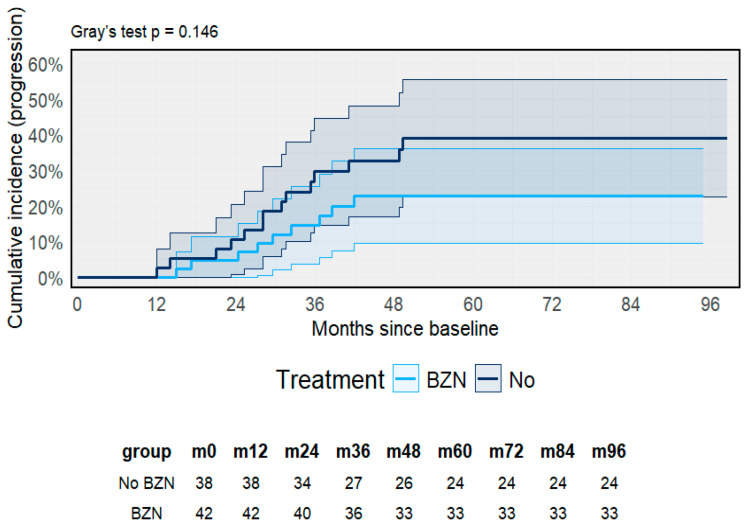
Cumulative incidence of clinical progression by treatment status. Fine–Gray competing-risk model with death as competing event (Gray’s test *p* = 0.146). Shaded area represents the 95% CI.

When accounting for death as a competing event, Fine–Gray sub-distribution models yielded similar results (Gray’s test *p* = 0.146) (Figure 7). The cumulative incidence of progression was consistently lower in the BZN group across follow-up, reaching approximately 25% compared with 35% among untreated participants at eight years. Although this difference did not reach statistical significance, the overall trend suggested a lower risk of progression among those receiving therapy.

**Figure 8:**
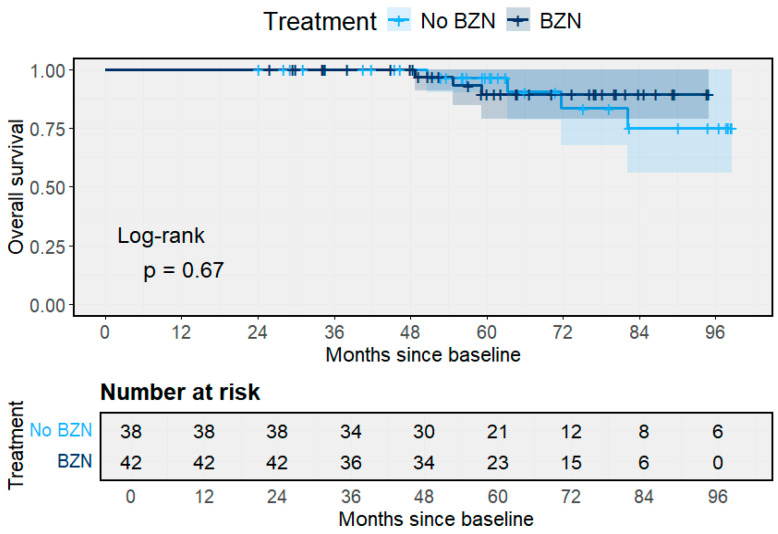
Overall survival according to Benznidazole treatment. Kaplan–Meier survival curves comparing treated and untreated participants (Log-rank *p* = 0.67). Shaded area represents the 95% CI.

Overall survival remained high and comparable between groups (Figure 8). At eight years, estimated survival was 91.8% among BZN-treated individuals and 89.4% among those without treatment (Log-rank *p* = 0.67). Deaths were infrequent and occurred mainly after five years of observation. The overlapping confidence intervals across groups indicate the absence of a measurable survival benefit attributable to treatment during the follow-up period.

## 4. Discussion

This long-term, community-based cohort offers one of the few prospective descriptions of the clinical evolution of chronic *T. cruzi* infection in a rural Colombian population living in a post-certification context. Following the successful interruption of intradomiciliary transmission, a growing number of individuals infected decades earlier are now reaching middle or older age, developing cardiac symptoms, and being diagnosed for the first time [23,24]. In this scenario, the cohort provides valuable evidence on the clinical course of chronic infection under conditions of surveillance and therapeutic access [18,19,23,24]. Despite the absence of new vector-borne infections, measurable rates of clinical progression and mortality were observed over eight years of follow-up, demonstrating that elimination of transmission does not equate to elimination of disease burden [25]. Approximately one in every ten participants experienced clinical deterioration, and overall mortality reached 9%, confirming that *T. cruzi* infection continues to have a long-term cardiovascular impact even in certified municipalities.

The incidence of progression from indeterminate to cardiac forms in this cohort was consistent with that reported in major longitudinal studies such as the Bambuí cohort in Brazil and the Cochabamba cohort in Bolivia, where annual incidence estimates range from 1 to 3 cases per 100 person-years, a pattern also observed in other Latin American countries [6,7,8,10,26,27]. These similarities indicate that, even after vector suppression, parasitic persistence and host-related factors continue to drive myocardial inflammation and fibrosis [28,29]. In Mogotes, no progression occurred among participants with normal ECG and ECHO and negative qPCR at baseline, emphasizing that the combination of electrical and molecular markers can effectively identify individuals at minimal risk of clinical evolution [20,30,31]. This approach is meant to strengthen post-certification surveillance systems by allowing more precise risk stratification and efficient resource allocation [32].

Multivariable modeling provides additional insight into individual determinants of progression. Baseline clinical stage was the strongest predictor, with hazards increasing stepwise from the indeterminate form to stages B2 and C–D, where the relative risk of worsening was particularly high. This gradient reinforces the prognostic importance of early subclinical myocardial involvement and supports the use of simplified staging systems for long-term monitoring in endemic areas [33]. Male sex and older age were also independently associated with progression, confirming patterns seen in Brazilian, Argentine, and Bolivian cohorts, where male predominance and cumulative cardiac stress have been linked to faster myocardial deterioration [9,10,34,35,36]. Together, these findings suggest that biological vulnerability and demographic aging converge to sustain the burden of chronic Chagas cardiomyopathy in post-elimination settings [36].

Interestingly, baseline ECG and echocardiographic abnormalities were not independently associated with progression once stage and demographic variables were included, possibly reflecting the small number of events and collinearity with clinical classification. Similarly, baseline qPCR positivity and left ventricular ejection fraction did not reach statistical significance, although both showed effect estimates consistent with higher risk [11,30]. These results indicate that, within clinically stratified populations, the prognostic weight of molecular and functional markers may vary according to baseline cardiac status. Continued molecular follow-up could help determine whether transient parasitemia plays a role in triggering micro-inflammatory episodes that contribute to gradual myocardial remodeling [33,37,38,39].

When BZN therapy was modeled as a time-dependent covariate, the association with reduced risk of progression persisted but did not reach statistical significance. The absence of a measurable effect is likely to reflect the limited sample size and event frequency rather than the absence of therapeutic benefit. The direction and magnitude of the hazard ratio are consistent with findings from the Bambuí and SaMi-Trop cohorts and post hoc analyses of the BENEFIT trial, all showing a trend toward reduced progression among treated participants [6,10,40,41,42]. These observations strengthen the growing consensus that etiological treatment may help delay or mitigate disease worsening even in chronic infection [7,41]. Expanding access to antiparasitic therapy should therefore remain a key pillar of Chagas disease control and post-certification strategies [43,44].

The ecological and genetic background of this population adds further relevance. In contrast with the Southern Cone, where TcII, V, and VI lineages predominate [38,45,46], Colombian transmission has historically involved the *R. prolixus* and TcI genotypes [47,48,49,50]. Although TcI has traditionally been associated with milder cardiac phenotypes, several studies from Colombia and northern South America have shown that TcI can also produce significant cardiomyopathy under chronic infection [51,52,53,54]. The persistence of progression in this context indicates that genotype alone does not determine outcome. Host immune response, reinfection history, comorbid conditions, and socioeconomic constraints are likely to interact when shaping the heterogeneous clinical trajectories observed [11,30,55,56]. These factors emphasize the importance of integrating Chagas care into primary healthcare and rural health equity policies within a One Health framework [57]. Notably, as TcI can be transmitted through both the sylvatic and peridomestic transmission cycles involving diverse mammalian reservoirs and triatomine vectors [45], it has somewhat diversified into two distinct sublineages, including TcIdom (domestic cycle) and TcIsyl (sylvatic cycle) [20,21]. Furthermore, although the relationship is not clear, DTU diversity may influence clinical outcomes [38,45,54,58,59]. TcI infections have been associated with predominantly cardiac manifestations with variable severity, while TcII and related lineages (TcV, TcVI) showed an association with both cardiac and digestive complications [45]. However, the extent to which these clinical differences reflect true parasite virulence remains obscure. In our cohort, TcIdom and TcIsyl were found in 9 and 13 patients respectively. In this cohort, only 7 patients died, most of them associated with a known level of heart disease. This aligns with epidemiological patterns observed in other TcI-endemic regions [38].

The persistence of progression and parasitemia in a population living under vector-control certification challenges the current paradigm of elimination. Certification should not mark the end of Chagas programs but their transformation from entomological surveillance toward comprehensive chronic disease management [13,60]. Sustained post-elimination surveillance should include periodic ECG and ECHO screening, molecular monitoring for parasitemia, and guaranteed access to BZN therapy. Embedding these strategies in routine primary healthcare is essential to prevent the silent accumulation of advanced cardiomyopathy and to ensure continuity of care for those already infected [61]. Integrating human, vector, and environmental surveillance under a One Health governance model could sustain elimination gains while addressing the residual burden of chronic disease.

It is important to highlight several important limitations. The first one is that the wide confidence intervals reflect limited statistical precision due to our sample size, which also can explain the small proportion of progression events. Therefore, no definitive conclusions about the magnitude of associations can be drawn now, though the direction of effects remains informative. Second, the absence of association between baseline qPCR results and disease progression in our cohort appears to contrast with prior evidence [58,59,62]. This discrepancy may reflect differences in patient populations or disease stage at enrollment. Additionally, a single baseline measurement followed by yearly evaluations may not adequately capture the dynamic nature of parasitemia in chronic Chagas disease. More frequent longitudinal parasite load trajectories may be more informative for predicting clinical outcomes. Third, our annual molecular testing protocol may underestimate the true frequency of parasitemia, as intermittent parasite detection between sampling points could be missed. More frequent sampling would provide a more comprehensive characterization of parasite dynamics, though this must be balanced against practical and resource constraints.

On the other hand, the strengths of this study are its prospective, community-based design, standardized cardiac assessments, and integration of molecular and therapeutic data within a real-world rural context, providing one of the most complete views of chronic *T. cruzi* infection after the interruption of domestic transmission. Limitations include the relatively small number of progression events, which constrained statistical power for rare outcomes such as stroke or sudden death, and the annual frequency of molecular testing, which may have underestimated transient parasitemia. Although follow-up retention was high, some survivor bias cannot be excluded, and extrapolation to other Colombian eco-epidemiological settings should be made cautiously.

## 5. Conclusions

In conclusion, chronic *T. cruzi* infection in this long-term Colombian cohort continued to progress at measurable rates despite the interruption of vectorial transmission. Advancing clinical stage, male sex, and older age were independent predictors of clinical worsening, while Benznidazole therapy showed a directionally protective effect. These findings reaffirm that the success of vector elimination must be accompanied by sustained clinical and molecular surveillance, longitudinal cardiac monitoring, and equitable access to antiparasitic treatment. Aligning these measures will ensure that Colombia’s post-certification strategy achieves not only entomological success but also tangible reductions in chronic disease burden and mortality.

## Figures and Tables

**Table 1:** Baseline demographic, clinical, and cardiovascular characteristics of the study cohort.

Characteristic	*T. cruzi* Infection Status
	Chronic Phase (n = 80)
Age (y), mean ± SD	54.7 ± 15.7
Female sex, n (%)	54 (67.5)
Systolic BP (mmHg), mean ± SD	125.9 ± 9.2
Heart rate (beats/min), mean ± SD	64.8 ± 8.5
BMI (kg/m^2^), mean ± SD	27.6 ± 5.3
*T. cruzi* qPCR-positive, n (%)	37 (46.2)
Median parasitic load, (parasite equivalents/mL) [IQR]	1.34 [1–4]
TcI_SYL_, n (%)	8 (21.6)
TcI_DOM_, n (%)	13 (35.1)
Diabetes mellitus, n (%)	15 (18.5)
Systemic arterial hypertension, n (%)	30 (37.5)
Dyslipidemia, n (%)	12 (15.0)
Associated heart disease, n (%)	21 (26.5)
Family history of Chagas disease n (%)	35 (43.8%)
ECG abnormalities, n (%)	43 (53.7%)
ECHO abnormalities, n (%)	17 (21.2)
Educational attainment (years) > 12, n (%)	21 (26.5)

SD: Standard deviation; BP: Blood pressure; BMI: Body Mass Index; qPCR: quantitative Polymerase Chain Reaction; IQR: Interquartile Range; ECG: Electrocardiogram; ECHO: Echocardiogram; TcI_SYL_: Sylvatic cycle associated *T. cruzi* strain; TcI_DOM_: Domestic cycle associated *T. cruzi* strain.

**Table 2:** Predictors of clinical progression in chronic Chagas disease (Cox proportional hazards model, linear effects).

Variable	Bivariate Analysis		Multivariate Analysis	
	Unadjusted HR (95% CI)	*p*-Value	Adjusted HR (95% CI)	*p*-Value
Clinical form				
Indeterminate	Ref			
Stage A	4.55 (0.47–43.72)	0.19	20.38 (0.98–422.45)	0.052
Stage B1	1.72 (0.11–27.46)	0.702	7.63 (0.27–212.88)	0.221
Stage B2	24.75 (3.08–198.71)	0.003	90.50 (3.70–2210.98)	0.005
Stage C–D	7.73 (0.48–124.56)	0.149	35.49 (0.58–2154.75)	0.09
Sex				
Female	Ref			
Male	3.08 (1.07–8.87)	0.038	7.41 (1.32–41.64)	0.023
ECG altered				
No	Ref			
Yes	0.61 (0.21–1.76)	0.361	0.15 (0.02–1.13)	0.067
ECHO altered				
No	Ref			
Yes	6.59 (2.27–19.13)	<0.001	0.74 (0.03–18.28)	0.85
Baseline qPCR positive				
No	Ref			
Yes	1.57 (0.54–4.52)	0.404	1.89 (0.47–7.54)	0.365
Age (per 10 years)	1.40 (1.02–1.92)	0.037	1.39 (1.07–2.52)	0.031
LVEF (per 5% absolute)	0.84 (0.66–1.06)	0.139	1.62 (0.70–3.73)	0.255

Ref: Reference; HR: Hazard ratio; CI: confidence interval; ECG: electrocardiogram; ECHO: echocardiography; qPCR: quantitative Polymerase Chain Reaction; LVEF: left ventricular ejection fraction.

## Data Availability

The data that support the findings of this study are property of the Instituto Nacional de Salud (INS) of Colombia and are subject to institutional data-sharing regulations. Access to these data can be requested through the corresponding author, upon reasonable request and approval by the INS.
